# Case report: multiple *UGT1A1* gene variants in a patient with Crigler-Najjar syndrome

**DOI:** 10.1186/s12887-018-1285-6

**Published:** 2018-10-03

**Authors:** Linda Gailite, Dmitrijs Rots, Ieva Pukite, Gunta Cernevska, Madara Kreile

**Affiliations:** 1 Scientific Laboratory of Molecular Genetics, Riga Stradiņš University, Dzirciema Street 16, Riga, LV 1007 Latvia; 2 Children’s Clinical University Hospital, Vienibas gatve 45, Riga, LV 1004 Latvia

**Keywords:** CNS-I, CNS-II, UGT1A1

## Abstract

**Background:**

Inherited unconjugated hyperbilirubinemia is caused by variants in the gene *UGT1A1* leading to Gilbert’s syndrome and Crigler-Najjar syndrome types I and II. These syndromes are differentiated on the basis of UGT1A1 residual enzymatic activity and its affected bilirubin levels and responsiveness to phenobarbital treatment.

**Case presentation:**

In this report, we present a boy with Crigler-Najjar syndrome type II with high unconjugated bilirubin levels that decreased after phenobarbital treatment but increased in adolescence. Four different *UGT1A1* gene variants have been identified for this patient, of which one is novel (g.11895_11898del) most likely confirming diagnose molecularly.

**Conclusions:**

The presented case highlights the challenges encountered with the interpretation of molecular data upon identification of multiple variants in one gene that are causing different degree reducing effect on enzyme activity leading to several clinical conditions.

## Background

Inherited unconjugated hyperbilirubinemia is caused by pathogenic variants in the *UGT1A1* gene and, depending on the bilirubin levels, is categorized as Gilbert’s syndrome (GS; OMIM: 143500), Crigler-Najjar syndrome type I (CNS-I; OMIM: 218800), or Crigler-Najjar syndrome type II (CNS-II; OMIM: 606785); the total bilirubin levels range from 17.1–102.6 μmol/L, 102.6–342 μmol/L, and 342–769.5 μmol/L, respectively [1]. When not appropriately treated, CNS-I is an early lethal condition characterized by kernicterus caused by the absence of active UDP-glucuronosyltransferase 1A1 (UGT1A1). In the case of CNS-II, UGT1A1 activity is greatly reduced [1].

There are numerous reports on *UGT1A1* gene variants that are related to the aforementioned clinical conditions [2, 3]. The most common condition is GS which affects ~ 10% of Europeans. As there are cases where it is difficult to distinguish the disorders caused by pathogenic variants in the *UGT1A1* gene, genetic investigations are becoming increasingly important [4].

The patient described here has unconjugated hyperbilirubinemia that is responsive to phenobarbital treatment and carries four genetic variations in the *UGT1A1* gene, one of which is reported for the first time.

## Case presentation

The proband is a 17-year-old Caucasian male who first presented at the neonatal stage with prolonged jaundice with an unconjugated bilirubin level of 400 μmol/L without hemolytic anemia data. Following an uneventful pregnancy, the patient was the first child of non-consanguineous Caucasian parents. Due to jaundice and elevated indirect bilirubin levels, he was diagnosed with GS soon after delivery as he had the A(TA)7TAA allele in homozygous state (identified by fragment analysis as previously described [5]). During his childhood years, the patient did well. He periodically underwent phenobarbital treatment for the reduction of bilirubin levels associated with jaundice; the mean bilirubin levels achieved as the result of these treatments were 100–200 μmol/L. The patient’s parents had no complaints until puberty.

After reaching puberty, the boy became severely jaundiced and his bilirubin levels increased to 300–350 μmol/L. However, there were no other complaints, he was doing well in school and his weight and height parameters were according to his age. The boy was referred to a hepatologist and multiple investigations were carried out. No pathological findings were observed in the laboratory analysis and hepatitis viral markers were negative. Furthermore, there was no evidence of other inborn errors of metabolism, i.e. organic acidurias and amino acidurias or changes in the fatty acid profile.

The results of abdominal ultrasound and abdominal MRI were unremarkable. Procurement of a percutaneous liver biopsy revealed no pathological findings. Based on isolated elevation of indirect bilirubin levels from standard laboratory investigations, reduced glutathione (0.89 mmol/l; reference value 1.12–1.216 mmol/l), a glucuronide level in 24-h urine of up to 102 mg/ml (reference value 430–600 mg/ml), phenobarbital responsiveness, and no evidence of kernicterus (as the child had no complaints about his health condition), CNS-II was suspected.

To confirm the CNS-II diagnosis, bidirectional sequencing of five exons and exon/intron boundaries of the gene *UGT1A1* (OMIM: 191740) was performed using previously described primers [6] and a BigDye 3.0 kit (following the manufacturer’s protocol; Applied Biosystems, USA). The nomenclature of the identified variations was identified by using Mutalyzer (https://mutalyzer.nl/) and assessed sequentially against the Single Nucleotide Polymorphism Database (dbSNP; www.ncbi.nlm.nih.gov/SNP), Exome Aggregation Consortium (ExAC; exac.broadinstitute.org), ClinVar (http://www.ncbi.nlm.nih.gov/clinvar), and the *UGT1A1* variant database [3]. A search was performed with MEDLINE if the variation was not found in any of the aforementioned databases. The biological significance of observed nucleotide changes located at splice sites was assessed using the Human Splicing Finder (HSF) 3.0 [7] and MutationTaster [8].

Four different variants in the *UGT1A1* gene were identified in the patient: g.3664A > C (c.1352A > C, rs3755319); g.4963_4964TA[7] (c.-53_-52insTA, A(TA)7TAA, UGT1A1*28, rs8175347); g.5884G > T (c.864 + 5G > T, IVS1 + 5G > T); and g.11895_11898del (c.996+2_996+5del) (reference sequence NG_033238.1) .

**Fig. 1:**
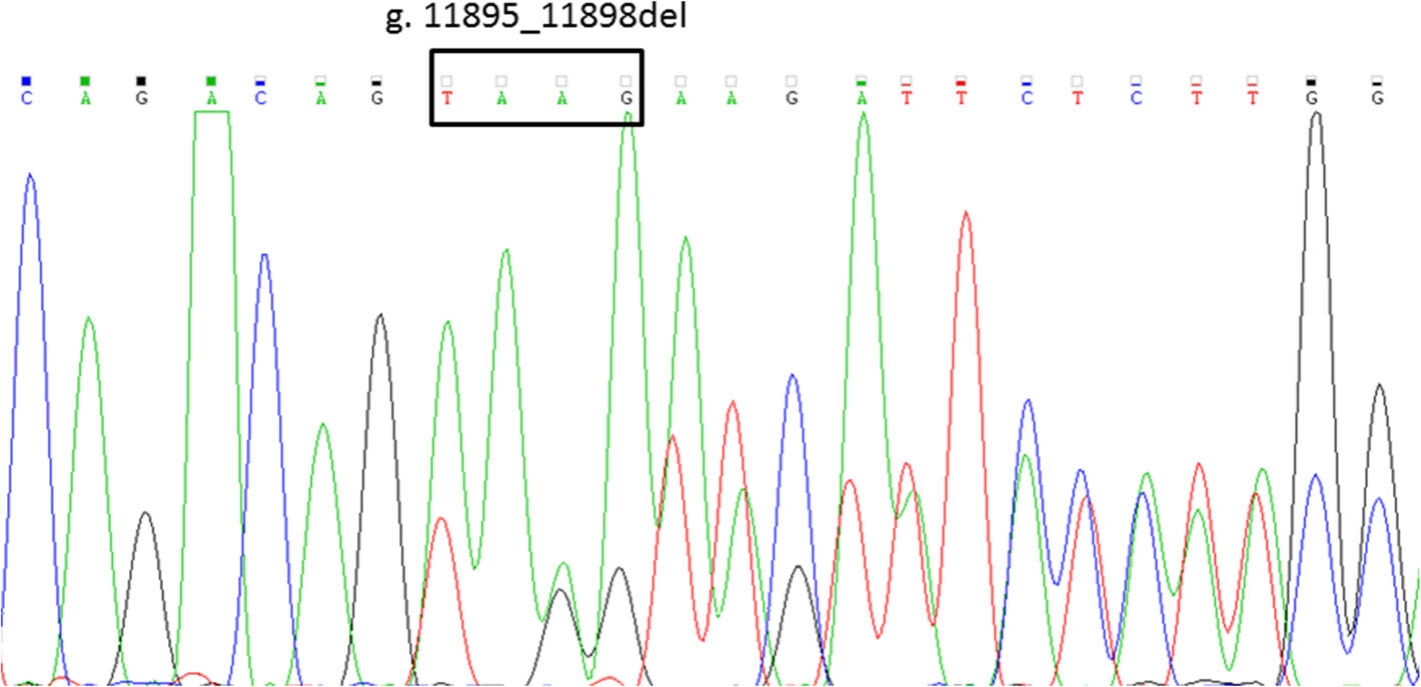
Electropherogram of the identified novel variant NG_033238.1: g.11895_11898del (c.996+2_996+5del) in case patient. In the first row showed reference sequence, in square given deleted nucleotides

**Fig. 2:**
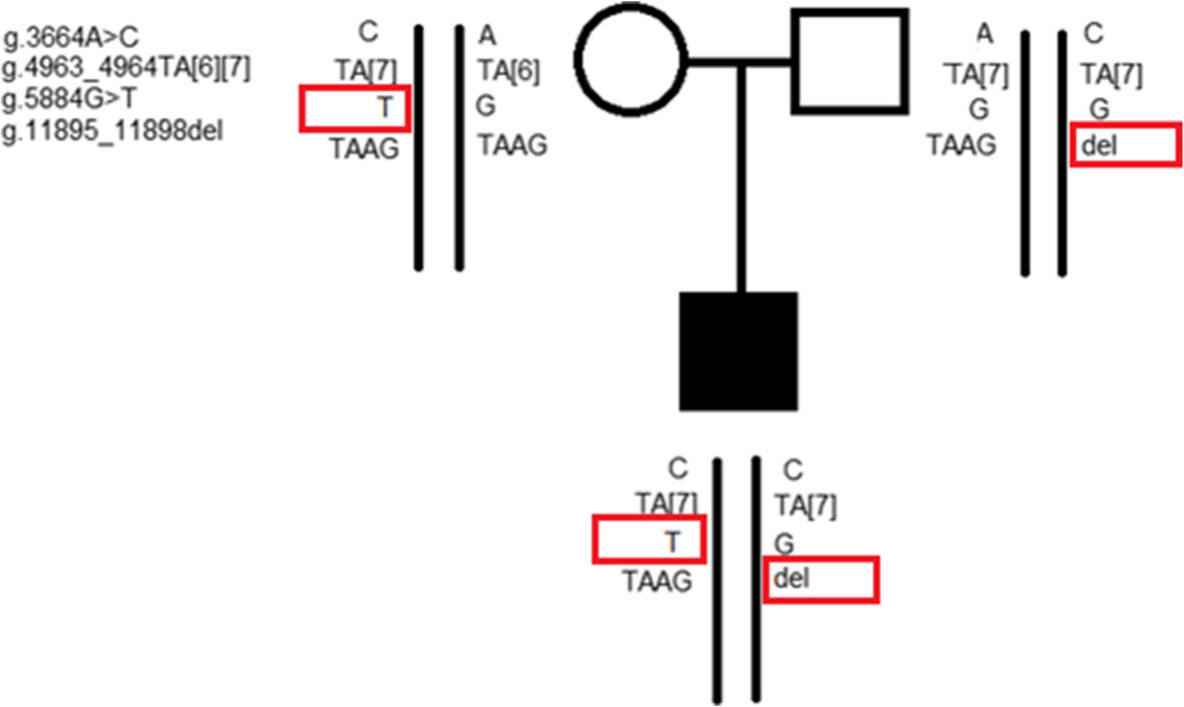
Pedigree of the family with Crigler-Najjar syndrome type II showing segregation of the identified genetic variants in the gene *UGT1A1* (reference sequence NG_033238.1)

In the ClinVar database, the variant g.4963_4964TA[7] is described as a variant affecting response to drug treatment. This is the most common variant identified in patients with GS. The variant g.3664A > C, as reported in the ClinVar database, causes transient familial neonatal hyperbilirubinemia (OMIM: 237900). The variant g.5884G > T located in the first intron has previously been reported in a patient with CNS-II [9], and from exome sequencing has been identified only in Europeans in five alleles (exac.broadinstitute.org). The second intronic variant g.11895_11898del (located in second intron two nucleotides after the second exon) is reported for the first time (sequence showed in the Fig. 1). Considering that one of the criteria for evaluating variant pathogenicity is its frequency in the healthy population, 90 healthy individuals from the Genome Database of the Latvian Population (LGDB), a government-funded biobank (the principle of LGDB has previously been reported [10]), were randomly selected and screened for the two lesser-described variants g.5884G > T and g.11895_11898del. The screened variants were not identified for any of the selected individuals. In order to identify variant segregation in the family, the parents were assessed for all the variants (Fig. 2). Both parents demonstrated normal-range levels of bilirubin from multiple measurements.

The intronic variants not mentioned in the ClinVar database were annotated according to ACMG standards and guidelines [11]. Both variants are classified as likely pathogenic (for variant g.5884G > T – fulfilled criteria PM2, PM3, PP3, PP4, PP5; for variant g.11895_11898del – fulfilled criteria PM2, PM3, PP3, PP4; Table 1).

**Table 1:** Pathogenicity criteria for two variants not reported in the ClinVar database according to ACMG standards and guidelines [11]

Evidence of pathogenicity	Fulfilled criteria with explanation	g.5884G > T	g.11895_11898del
Moderate evidence	PM2 – the prevalence of the variants in affected individuals is significantly increased compared with the prevalence in controls	1) The variant was not found in 180 healthy Latvian control chromosomes 2) 0.00004119 (for Europeans in ExAC database)	1) The variant was not found in 180 healthy Latvian control chromosomes 2) Not reported^a^
	PM3 – for recessive disorders, detected in trans with a pathogenic allele	Located in trans position with other allele (in our case likely pathogenic allele)	Located in trans position with other allele (in our case likely pathogenic allele)
Supporting evidence	PP3 – multiple lines of computational evidence support a deleterious effect on the gene product	1) HSF – predicted WT donor site broken ΔCT- -13.62%^b^ (max entropy − 67.38%^c^) 2) Mutalyzer – variant located near to splice site 3) MutationTaster – disease causing (protein features (might be) affected; splice site change)	1) HSF – predicted WT donor site broken ΔCT-55.3%^b^ (max entropy − 284.9%^c^) 2) Mutalyzer - variant located in splice site 3) MutationTaster – disease causing (protein features (might be) affected; splice site change)
	PP4 – patient’s phenotype or family history is highly specific for disease with single genetic etiology	Yes	Yes
	PP5 – reputable source recently reports variant as pathogenic, but the evidence is not available to the laboratory to perform an independent evaluation	Passuello et al., 2009	None
Total score		Likely pathogenic	Likely pathogenic

^a^As identified variant is indel frequency, should be evaluated with caution

^b^ ΔCT – if difference between consensus value for wild type and mutated variantis below − 10% it is considered that splice site is broken [7]

^c^if maximal entropy is below − 30%, it is considered that splice site is broken [7]

## Discussion and conclusions

Three inherited disorders leading to unconjugated hyperbilirubinemia have been identified. They are categorized depending on the bilirubin levels, phenobarbital responsiveness, and clinical picture.

We present here a 17-year-old male with CNS-II who has significant hyperbilirubinemia that decreases after phenobarbital intake and some UGT1A1 activity confirmed by glucuronide traces in his urine. He carries four genetic variants in the *UGT1A1* gene; one reported for the first time. Phenobarbital responsiveness and the absence of kernicterus are described as criteria to discriminate CNS-I and CNS-II [1, 12]. However, atypical cases have also been reported. Duhamel et al. described a 25-year-old male with total bilirubin levels suggestive of CNS-I but without any neurological changes and phenobarbital unresponsiveness; no molecular investigations were carried out for that particular patient [13]. Chalasani et al. reported a CNS-II patient who developed kernicterus after laparoscopic surgery, leading to hematoma and resulting in a more significant increase in bilirubin production; the patient had three mutations: A(TA)7TAA in homozygous state and c.1391A > C in heterozygous state [14].

With regard to genotype/phenotype correlation in the case of variants of *UGT1A1*, Kadakol et al. proposed how to differentiate between CNS-I and CNS-II based upon molecular findings [15]. CNS-I results from genetic lesions that cause the premature truncation of UGT1A1, also caused by splice defects or substitution of critical amino acid residues, whereas CNS-II is caused by the substitution of one amino acid residue that markedly reduces without abolishing the catalytic activity of the enzyme [15]. Li et al. analyzed CNS-II patients in the Chinese population and discovered the existence of a genotype/phenotype correlation [16]. Patients homozygous for both variations G71R and Y486D had a bilirubin level > 200 μmol/L, but patients with total bilirubin levels ranging from 102.6 to 200 μmol/L carried a broader spectrum of *UGT1A1* variants [16]. However, due to a different variant spectrum, these data are not attributable to Europeans.

Our described patient has four different molecular variants. This is the first reported case of four variants in one patient; previous reports have identified three variants in a single patient [17–19]. It is not clear how multiple variants in the *UGT1A1* gene affect the clinical status. Kadakol et al. described four different gender and age families with identified multiple genetic variants in patients with unconjugated hyperbilirubinemia (> 100 μmol/L) and concluded based on the high frequency of *UGT1A1* promoter variant A(TA)7TAA that further examination of the structural variants in the gene should be conducted for patients with hyperbilirubinemia as it could help find more severe phenotypes [15]. The variant A(TA)7TAA in homozygous state leads to 70% reduction in UGT1A1 expression compared to A(TA)6TAA carriers [20], and the presence of additional variants is likely to affect the enzyme activity even more [15].

Our patient has the rs3755319 variant that could explain his neonatal hyperbilirubinemia, the A(TA)7TAA variant that could contribute to his prolonged hyperbilirubinemia, and two other variants, one of which (c.864 + 5G > T) has been reported in a compound heterozygous state in pregnant women with variant c.1175C > T in patients with CNS-II [9]. The other variant (g.11895_11898del) is reported for the first time and according to the ACMG guidelines could be classified as a likely pathogenic variant (see Table 1). Unfortunately, no functional studies for the identified variants have been performed as UGT1A1 is expressed only in the liver [21] and there were no indications for a liver biopsy, no RNA sample was available and in vitro study as presented by Gupta et al. were not performed [22]. Splice site mutations in *UGT1A1* were first reported in CNS-I [23], then reported in a subsequent mutation classification to be causing CNS-I [2]. However, although the total bilirubin levels of our patient in adolescence correspond to CNS-I, he is responsive to phenobarbital treatment and to date there are no signs of kernicterus, thus classifying as CNS-II. According to the ACMG guidelines, functional studies provide likely pathogenic criteria for the interpretation of the classification of unreported splice site variants. In the case of our patient, functional studies were not conducted. However, previous studies have shown that functional studies correlate well with pathogenicity prediction tools in case of *UGT1A1* allelic variants [22], but to our knowledge there is no computational tool for analysing multiple allelic variant effect on one allele. In our study computation tools were used analysing effect of single allelic variant – here, MutationTaster and HSF predicted that the variation breaks the wild type splice site and as the variant is localized in the intron after exon 2, then it would affect all transcripts of the gene. Also based on family segregation analysis clinical symptoms could be caused by both variants g.11895_11898del and c.864 + 5G > T located in trans position, because for both parents one variant was identified but without remarkable changes in the bilirubin level.

The reported case demonstrates that it is not always possible to distinguish between CNS-I and CNS-II based on molecular findings and for patients with elevated unconjugated bilirubin level if confirming most common genotype for GS (homozygote for (A(TA)7TAA allele) should be performed full *UGT1A1* sequencing if level of unconjugated bilirubin is atypically high Furthermore, wider opportunities present a greater challenge concerning interpretation, as encountered by our group when identifying an unreported variant.

Study limitation is missing functional studies of multiple variant effects on UGT1A1 expression level.

## Abbreviations

ACMG: American College of Medical Genetics
CNS-I: Crigler-Najjar syndrome type I
CNS-II: Crigler-Najjar syndrome type II
CT: Consensus value
dbSNP: Single Nucleotide Polymorphism Database
ExAC: Exome Aggregation Consortium
GS: Gilbert’s syndrome
HSF: Human Splicing Finder
LGDB: Genome Database of the Latvian Population
MRI: Magnetic resonance imaging
OMIM: Online Mendelian Inheritance in Man
PM: Moderate evidence of pathogenicity
PP: Supporting evidence of pathogenicity
UGT1A1: UDP-glucuronosyltransferase 1A1
WT: Wild type
